# Correlation of the two most frequent HLA haplotypes in the Italian population to the differential regional incidence of Covid-19

**DOI:** 10.1186/s12967-020-02515-5

**Published:** 2020-09-15

**Authors:** Simona Pisanti, Joris Deelen, Anna Maria Gallina, Mariella Caputo, Marianna Citro, Mario Abate, Nicoletta Sacchi, Carmine Vecchione, Rosanna Martinelli

**Affiliations:** 1 Department of Medicine, Surgery and Dentistry ‘Scuola Medica Salernitana’, University of Salerno, Via Salvatore Allende, 84081 Baronissi, SA Italy; 2 Max Planck Institute for Biology of Ageing, PO Box 41 06 23, 50866 Cologne, Germany; 3 E.O. Galliera, Italian Bone Marrow Donor Registry, Genoa, Italy; 4 Vascular Pathophysiology Unit, IRCCS Neuromed, Via Atinense, Pozzilli, 86077 Isernia Italy

**Keywords:** HLA polymorphisms, Covid-19 incidence and mortality, Susceptibility, HLA typing

## Abstract

**Background:**

Understanding how HLA polymorphisms may affect both susceptibility, course and severity of Covid-19 infection could help both at the clinical level to identify individuals at higher risk from the disease and at the epidemiological one to explain the differences in the epidemic trend among countries or even within a specific country. Covid-19 disease in Italy showed a peculiar geographical distribution from the northern most affected regions to the southern ones only slightly touched.

**Methods:**

In this study we analysed the regional frequencies for the most common Italian haplotypes from the Italian Bone Marrow Donor Registry (HLA-A, -B, -C and -DRB1 at four-digit level). Then we performed Pearson correlation analyses among regional haplotypes estimated frequency in the population and Covid-19 incidence and mortality.

**Results:**

In this study we found that the two most frequent HLA haplotypes in the Italian population, HLA-A*:01:01g-B*08:01 g-C*07:01g-DRB1*03:01g and HLA-A*02.01g-B*18.01g-C*07.01g-DRB1*11.04g, had a regional distribution overlapping that of Covid-19 and showed respectively a positive (suggestive of susceptibility) and negative (suggestive of protection) significant correlation with both Covid-19 incidence and mortality.

**Conclusions:**

Based on these results, in order to define such HLA haplotypes as a factor effectively associated to the disease susceptibility, the creation of national networks that can collect patients’ samples from all regions for HLA typing should be highly encouraged.

## Background

The novel coronavirus identified in the last months of 2019 (SARS-CoV-2) belongs to the family of already known human CoVs of zoonotic origin, along with 229E, OC43, HKU1, NL63, that are community acquired CoVs, well adapted to humans, causing mild respiratory diseases. The CoVs causing severe acute respiratory syndrome (SARS-CoV) and Middle East respiratory syndrome (MERS-CoV), that are on the contrary highly pathogenic and cause severe respiratory disease with significantly high case fatality (9.6% for SARS-CoV and 34.4% in MERS-CoV), also belong to this family [1]. SARS-CoV-2-induced pneumonia, named by World Health Organization as coronavirus disease 2019 (Covid-19), has been declared a pandemic on the 11th of March 2020 since its first appearance in Wuhan, China, in December 2019 [2]. Italy was the first European country to report an outbreak of infections with two hot spots located in the northern of Italy, which led to the Lombardia and Veneto regions being defined as red zone, followed by complete isolation of these areas from the 21th of February onward. By that time, the epidemic had spread all over Italy and the lockdown was extended to the entire country on the 9th of March to further limit the diffusion and avoid the collapse of the public health system [3]. Since then, for many weeks Italy has been the country with the highest number of cases and deaths worldwide. With the pandemic evolving it has settled in sixth place for the number of confirmed infections in the world (more than 233,000) and third for the number of deaths (more than 33,300) as of the 31st of May (https://coronavirus.jhu.edu/map.html) [4]. These numbers could even be an underestimation, because of hidden asymptomatic or pauci-symptomatic individuals not subjected to the control swab. Even death counts could have been underreported especially in the climax of the emergency, as supported by several studies and by a report analysis of mortality in the period of epidemic from Covid-19 by National Institute for Social Security (INPS) [5, 6]. During this time the assistance and monitoring network was unprepared to face a pathology still unknown in many respects, hospitals and intensive care units were overcrowded and many people may have died in their homes without testing. A recent observational study conducted on a small community in Nembro, a little town located in Lombardia, one of the most affected areas of northern Italy, reported an all-cause mortality between January and April 2020, several times higher than that recorded in the previous 8 years in the same time frame, reaching a peak of 154.4 per 1000 person years in March 2020 *vs* a range 1.0 to 21.5 per 1000 person years between January 2012 and February 2020 [5]. Overall, Covid-19 deaths were mostly observed in males and older patients with pre-existing comorbidities [7, 8]. However, the still inexplicable high Case Fatality Rate that has been reported in Italy compared to other countries for the age group over 60, has not proven relationship with the demographic characteristics and the percentage in the elderly population [9, 10].

Even though Covid-19 pathogenesis has not yet been fully disclosed, the host antiviral response undoubtedly plays a key role in the disease course. Immunopathogenesis and induction of a proinflammatory cytokine storm are the key event in disease progression into severe forms leading to acute respiratory distress syndrome (ARDS). When the adaptive immune response fails to clear the infection from the host, the disease progresses to more severe stages since the virus rapidly spreads into different organs (lungs, intestine, kidney) eliciting a massive tissue destruction and a strong inflammatory response. These are mediated by innate immune cells, mainly macrophages and granulocytes, that induce a severe or even fatal clinical outcome caused by multi-organ dysfunction, through a cytokine storm that spreads throughout the body [11]. High systemic levels of IL-6, IL-7, TNFα, IL-10, G-CSF, MCP-1 and MIP1α have been observed in the blood of Covid-19 patients, with a correlation to disease severity [12].

A fundamental question that urgently deserves an answer is why, even considering only symptomatic patients, the disease progresses into a severe form compromising respiratory function only in a fraction of the infected individuals. One factor could be the appropriateness of the immune response to elicit a specific antiviral immunity without destroying the host tissues, which depends both on environmental and genetic factors. In this context, the human leukocyte antigen (HLA) complex, which is well known to influence the efficacy of T cell recognition of foreign antigens, could play a major role. The presentation of viral antigens through HLA II by APC cells is a key event in the establishment of the anti-viral adaptive immune response in addition to HLA I direct presentation to cytotoxic CD8 T cells. The HLA locus is the most polymorphic region in the human genome. The polymorphism of HLA proteins controls the possible repertoire of bound epitopes, thus shaping the immune response profile of an individual [13, 14]. Genetic polymorphisms have been reported to influence population and individual predisposition to multifactorial, autoimmune and infectious pathologies [15]. Susceptibility to viral infections like human immunodeficiency virus (HIV), human hepatitis B virus (HBV), human hepatitis C virus (HCV) and human papilloma virus (HPV), to name just a few, has been reported to be influenced by HLA specific subtypes [16–19]. Noteworthy, studies conducted on a meaningful data set of high-resolution HLA-typed individuals, revealed significant differences in single allele and haplotypes frequencies among the Italian regions [20]. Understanding how genetic variation in HLA may affect both susceptibility as well as course and severity of Covid-19 infection, could help to identify and stratify individuals at higher risk from the disease. Moreover, it could help to give a possible explanation at the epidemiological level why the epidemic in Italy, even though spread all over the country, showed a strong regionality with northern regions, above all Lombardia, reporting higher rates than central and southern regions.

On these premises, in this work we performed a geographical epidemiological analysis in order to find if in the Italian population there are particular frequent haplotypes and HLA alleles, whose distribution among the Italian regions overlaps with Covid-19 regional distribution and thus formulate and test the hypothesis of their potential association with Covid-19 incidence and severity, to identify sub-populations most at risk of susceptibility to the infection.

## Methods

### Data sources

For our analyses we used the large dataset from the Italian Bone Marrow Donors Registry (IBMDR) maintained at E.O. Ospedali Galliera di Genova https://www.ibmdr.galliera.it/ibmdr/ [20]. In the study, we used the dataset 2, which is constituted by a sample of 104,135 donors with available data about the city of birth, typed for HLA-A, -B, -C and -DRB1 at a high-resolution (HR) level by ASHI or EFI-accredited tissue typing laboratories using HR molecular biology techniques (SBT, SSO, SSP, NGS) as described [20]. The data, based on the donor’s birth region, were divided into the 20 geographical regions of Italy that are in alphabetical order: Abruzzo, Basilicata, Calabria, Campania, Emilia Romagna, Friuli Venezia Giulia, Lazio, Liguria, Lombardia, Marche, Molise, Piemonte, Puglia, Sardegna, Sicilia, Toscana, Trentino Alto Adige, Umbria, Valle d’Aosta and Veneto. The datasets used as reference are the CWD 2.0.0 catalogue (ASHI CWD) from worldwide population and the EFI CWD catalogue for the European population [21, 22]. Sardegna and Valle D’Aosta regions were excluded from correlation analyses because of their widely recognized genetic difference, even within the HLA locus, with respect to the rest of Italy due to genetic isolation as previously reported [23].

With regard to the number of Covid-19 cases and deaths, we used the data collected by the Italian National Institute of Health (Istituto Superiore di Sanità [ISS]), which are daily reported by the Civil Protection Department Headquarters and published at http://www.salute.gov.it.

Moreover, we obtained data on the number of inhabitants for each Italian region that is freely available from the Italian National Institutes of Statistics (ISTAT), the main provider of official statistics in Italy, for both citizens and policy-makers.

### Statistical analysis

All statistical analyses were performed using R version 4.0.0 (R Core team) [24]. Pearson correlations, as measure of the strength of the linear relationship between two variables (− 1 < r ≤ + 1) and accompanying P-values, were calculated using the package ‘Hmisc’. Correlation plots were generated using the package ‘ggpubr’. P values were considered statistically significant below 0.05 (* < 0.05, ** < 0.01, *** < 0.001).

## Results

### Geographical distribution of Covid-19 epidemic in Italy

**Fig. 1:**
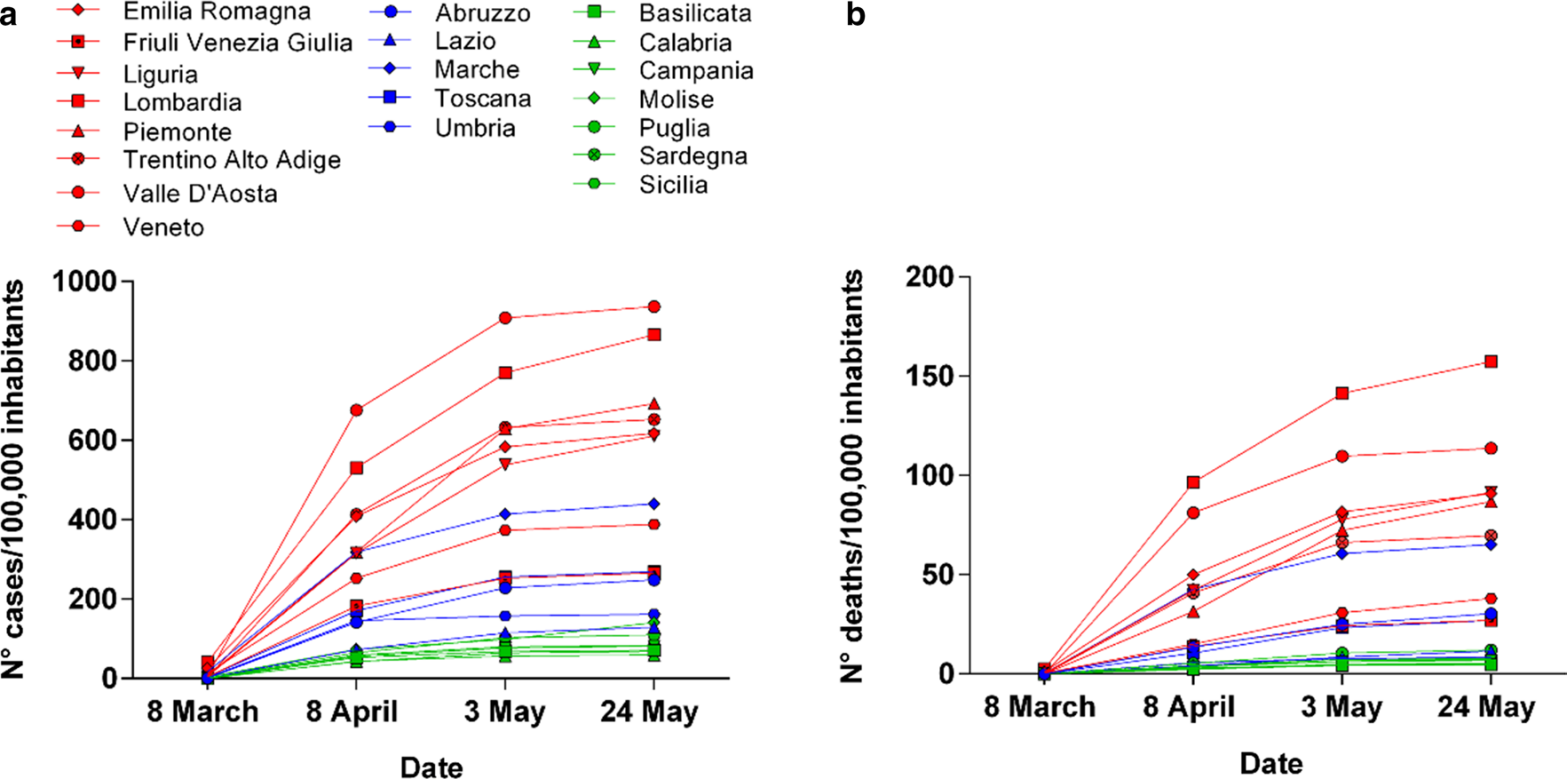
Trend over time relative to the number of Covid-19 cases/100,000 inhabitants and deaths/100,000 inhabitants. The graphs report the number of Covid-19 cases/100,000 inhabitants (**a**) and deaths/100,000 inhabitants (**b**) at four time points of the epidemic. Red symbols are used for northern regions, blue symbols for central regions and green symbols for southern regions

We analysed the number of Covid-19 cases confirmed by a real-time reverse transcriptase–polymerase chain reaction (RT-PCR) assay of nasal and pharyngeal swabs from patients and the number of deaths reported by ISS for each Italian region. The analysis was performed at four meaningful time points of the epidemic: before the lockdown start (8th of March), 1 month later during the exponential phase of the epidemic (8th of April), at the end of the lockdown (3rd of May) and 3 weeks later (24th of May) (Table 1). The values for the number of cases and deaths were normalised to the total of inhabitants of each region, in order to take into account the different population sizes, based on statistics reported by ISTAT for 2019 (Additional file 1: Table S1). At every time point, there is a clustering of the twenty regions for number of cases and deaths in three groups reflecting the geographical localization, with the northern regions showing the most cases and deaths (Fig. 1).

**Table 1:** Regional data relative to the impact of COVID-19 on the Italian population

Region	Macroregion	8 March		8 April		3 May		24 May	
		N° cases/100,000 inhabitants	N° deaths/100,000 inhabitants	N° cases/100,000 inhabitants	N° deaths/100,000 inhabitants	N° cases/100,000 inhabitants	N° deaths/100,000 inhabitants	N° cases/100,000 inhabitants	N° deaths/100,000 inhabitants
Abruzzo	Central	1.30	0.00	141.74	13.65	228.43	25.16	249.01	30.35
Basilicata	South	0.71	0.00	52.77	2.49	68.58	4.44	70.89	4.80
Calabria	South	0.46	0.00	44.12	3.08	57.21	4.52	59.42	4.93
Campania	South	1.74	0.00	56.33	3.81	77.29	6.27	81.86	6.98
Emilia Romagna	North	26.46	1.26	408.88	50.10	583.39	81.67	617.96	90.93
Friuli Venezia Giulia	North	4.69	0.08	182.52	13.91	252.79	24.44	266.29	27.07
Lazio	Central	1.48	0.05	72.56	4.15	115.82	8.64	129.73	11.63
Liguria	North	5.03	0.39	316.39	42.18	539.07	77.97	611.36	91.51
Lombardia	North	41.64	2.65	530.92	96.63	770.61	141.45	865.86	157.45
Marche	Central	17.83	0.46	318.57	42.75	414.29	60.78	440.18	65.17
Molise	Central	4.58	0.00	73.95	4.25	98.49	7.20	141.35	7.20
Piemonte	North	8.26	0.11	318.68	31.63	629.65	72.35	692.77	86.84
Puglia	South	0.99	0.07	65.38	5.44	102.85	10.52	110.65	12.09
Sardegna	South/Island	0.67	0.00	59.47	3.60	80.45	7.26	82.70	7.87
Sicilia	South/Island	1.06	0.00	43.18	2.66	64.80	4.84	68.46	5.38
Toscana	Central	4.45	0.00	171.04	10.51	256.41	23.38	269.78	27.16
Trentino	North	2.98	0.00	413.79	40.85	632.58	66.21	652.54	69.76
Umbria	Central	2.95	0.00	146.14	5.67	158.05	7.71	162.13	8.50
Valle D’Aosta	North	7.16	0.00	676.40	81.17	908.76	109.81	937.41	113.79
Veneto	North	13.66	0.37	252.96	15.00	373.39	30.90	389.05	38.10

### Regional distribution of most frequent HLA haplotypes

**Fig. 2:**
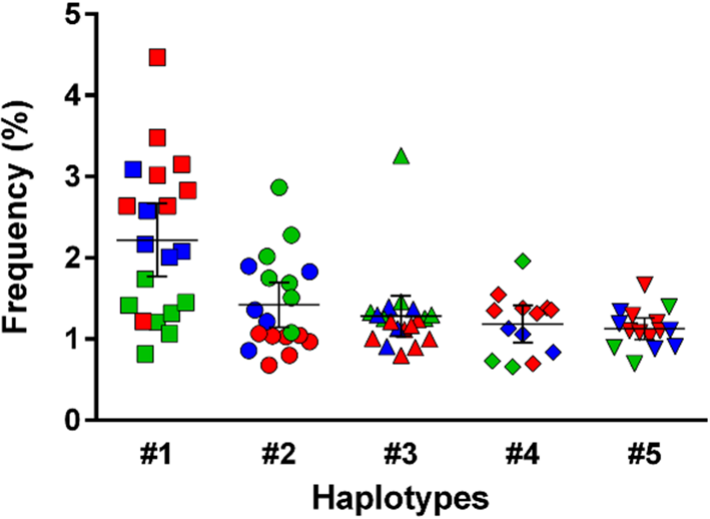
Frequencies of the 5 most common haplotypes observed in the Italian population. The horizontal bars indicate the mean national values plus the 95% confidence interval. The # refers to the ranking of the haplotype for frequency in the Italian population. Red symbols are used for northern regions, blue symbols for central regions and green symbols for southern regions

Given the key role of the host immune response against the SARS-CoV-2 virus in the pathogenesis of the disease and the high degree of HLA polymorphism, we subsequently tried to determine, at the general population level, if there are significative differences in the frequency of the most frequent HLA haplotypes in the Italian population among the northern, central and southern regions. We performed our analyses on the five most common Italian haplotypes as ranked by the Italian Bone Marrow Donor Registry (IBMDR), the most extensive Italian collection consisting of more than 131,000 high resolution HLA-A, -B, -C and -DRB1 typed individuals at the four-digit level. The registry contains complete information about the region of provenience and ethnic origin for a sample of 104,135 subjects, thus providing a reliable estimation of HLA frequencies within the Italian population [20]. The estimated national frequencies for these haplotypes, calculated using the Arlequin programme by the EM algorithm, sum up to 6.9%: HLA-A*:01:01g-B*08:01g-C*07:01g-DRB1*03:01g (2.54%); HLA-A*02.01g-B*18.01g-C*07.01g-DRB1*11.04g (1.14%); HLA-A*30.01g-B*13.02g-C*06.02g-DRB1*07.01g (1.09%); HLA-A*29.02g-B*44.03g-C*16.01g-DRB1*07.01g (1.08%); HLA-A*03.01g-B*07.02g-C*07.02g-DRB1*15.01g (1.02%). The regional frequencies estimated from the data set sample are depicted in Table 2. We observed that the most frequent five Italian haplotypes were not uniformly distributed in all regions and, in some regions, they were totally missing. Sardegna and Valle D’Aosta regions which are widely recognized for their genetic difference in the HLA locus with respect to the rest of Italy due to genetic isolation, even if reported in the Tables to have an overall picture of the geographic distribution of HLA haplotypes in the Italian population, have been indeed excluded from all subsequent correlation analyses [23]. The haplotypes ranked#1 HLA-A*01:01g-B*08:01g-C*07:01g-DRB1*03:01g and #2 HLA-A*02:01g-B*18:01g-C*07:01g-DRB1*11:04g showed the highest dispersion from the mean national value and an almost net clustering among northern, central and southern regions in the opposite direction for #1 and #2 (Fig. 2).

**Table 2:** Frequencies of the 5 most common haplotypes observed in the Italian population

Region	Frequencies (%)				
	#1 HLA-A*:01:01g-B*08:01g-C*07:01g-DRB1*03:01g	#2 HLA-A*02.01g-B*18.01g-C*07.01g-DRB1*11.04g	#3 HLA-A*30.01g-B*13.02g-C*06.02g-DRB1*07.01g	#4 HLA-A*29.02g-B*44.03g-C*16.01g-DRB1*07.01g	#5 HLA-A*03.01g-B*07.02g-C*07.02g-DRB1*15.01g
Italy	2.54	1.14	1.09	1.08	1.02
Abruzzo	2.08	1.90	1.40	0.00	1.34
Basilicata	1.21	2.87	1.25	0.00	1.40
Calabria	0.82	1.75	1.26	0.00	0.00
Campania	1.42	1.69	1.33	0.73	0.70
Emilia Romagna	2.64	0.97	1.12	1.55	1.29
Friuli Venezia Giulia	3.15	0.80	0.80	0.70	1.10
Lazio	2.01	1.36	1.13	0.84	1.19
Liguria	3.02	1.05	1.24	1.35	1.20
Lombardia	2.83	1.03	1.01	1.38	1.03
Marche	2.58	1.83	1.30	0.00	0.88
Molise	1.74	2.02	3.26	1.96	0.00
Piemonte	2.64	1.04	0.90	1.32	1.10
Puglia	1.32	2.28	1.46	0.00	0.90
Sardegna	1.07	1.08	0.00	0.00	0.00
Sicilia	1.45	1.51	1.30	0.66	0.00
Toscana	3.09	0.86	1.37	1.13	1.11
Trentino Alto Adige	4.47	0.68	1.17	1.38	1.67
Umbria	2.17	1.22	0.91	1.06	0.91
Valle D’Aosta	1.22	0.00	1.22	0.00	0.00
Veneto	3.48	1.07	1.00	1.36	1.08

The # indicates the ranking of the haplotype for frequency in the Italian population

### Correlation among HLA haplotypes regional frequency and Covid-19 incidence and mortality

**Fig. 3:**
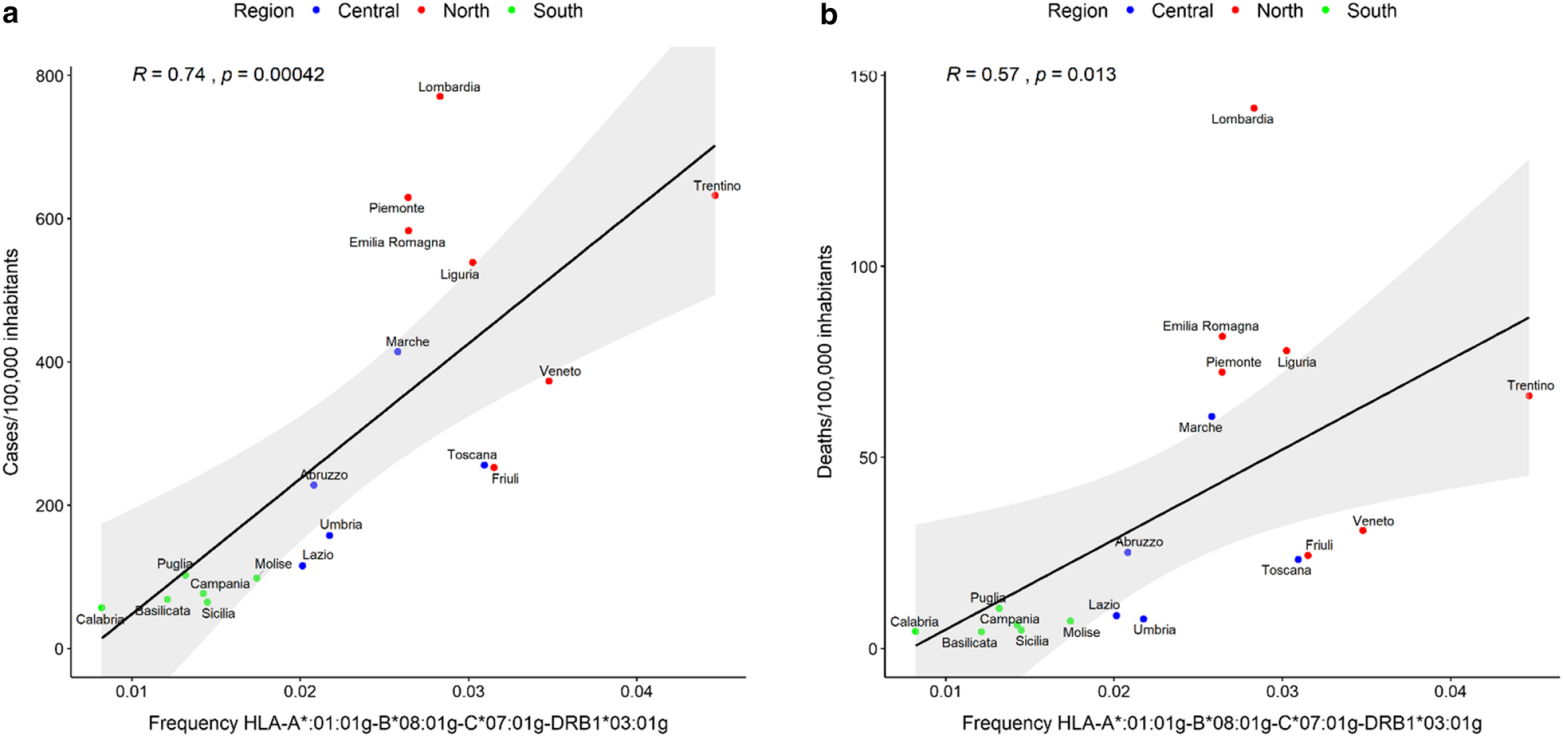
Bivariate correlation analysis among the regional frequency of HLA-A*01:01g-B*08:01g-C*07:01g-DRB1*03:01g haplotype and the N° cases/100,000 inhabitants and N° deaths/100,000 inhabitants. The graphs show the bivariate correlation analysis relative to 3rd May time point. High HLA-A*01:01g-B*08:01g-C*07:01g-DRB1*03:01g frequency in the population is significantly correlated with a high number of both cases (**a**) and deaths (**b**)/100,000 inhabitants

**Fig. 4:**
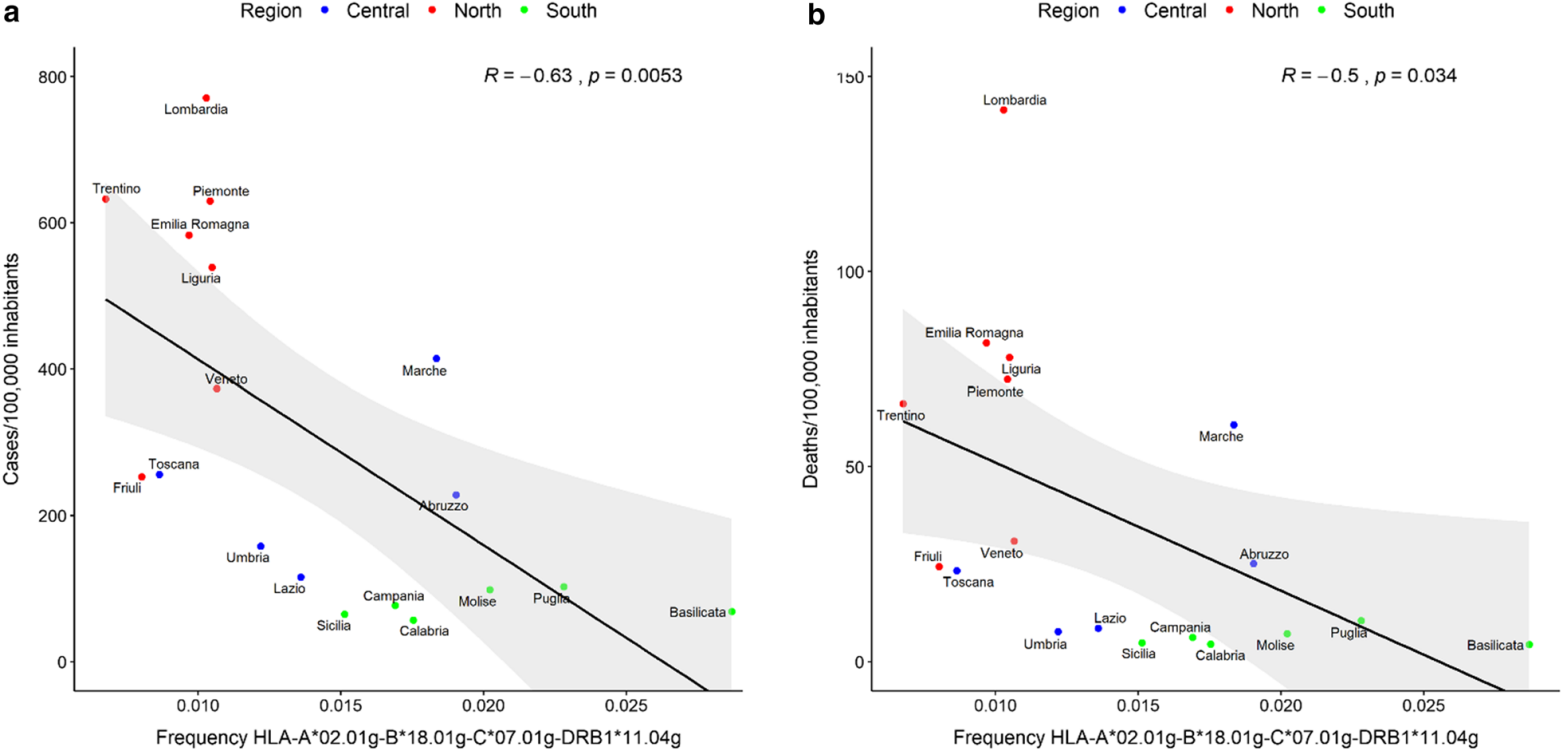
Bivariate correlation analysis among the regional frequency of the HLA-A*02.01g-B*18.01g-C*07.01g-DRB1*11.04g haplotype with the N° cases/100,000 inhabitants and N° deaths/100,000 inhabitants. The graphs show the bivariate correlation analysis relative to 3rd May time point. High HLA-A*02.01g-B*18.01g-C*07.01g-DRB1*11.04g frequency in the population is significantly correlated with a low number of both cases (**a**) and deaths (**b**)/100,000 inhabitants

Next, in order to find if there is an overlap among the most frequent haplotypes distribution and the incidence of Covid-19 at the regional level, we calculated, using Pearson correlations, if and how the regional frequencies of each haplotype in the population linearly correlate with the regional number of both Covid-19 cases and deaths/100,000 inhabitants. We found that the haplotype ranked #1 HLA-A*01:01g-B*08:01g-C*07:01g-DRB1*03:01g shows a positive (suggestive of susceptibility) significant correlation with both Covid-19 incidence and mortality. Conversely, the haplotype ranked #2 HLA-A*02:01g-B*18:01g-C*07:01g-DRB1*11:04g shows a negative correlation (suggestive of protection). This correlation is observed at all significant time points of the epidemic except for the 8th of March when the numbers were still too low. Pearson’s correlation coefficients and relative P values for each bivariate analysis are reported in Table 3. For the haplotype #1 HLA-A*01:01g-B*08:01g-C*07:01g-DRB1*03:01g, the distribution is characterized by a net clustering of the regions in three groups, with the northern regions reporting high frequency values and corresponding highest incidence and mortality, the central regions displaying intermediate values and the southern regions the lowest values for the haplotype #1 (Fig. 3). The Pearson correlation coefficient among the frequency of HLA-A*01:01g-B*08:01g-C*07:01g-DRB1*03:01g haplotype and Covid-19 N° cases/100,000 inhabitants ranges from 0.34 (at the 8th of March) to 0.75 (at the 8th of April). When considering the N° of Covid-19 deaths/100,000 inhabitants as the correlated variable, the Pearson’s coefficient varies from 0.24 to 0.57 (Table 3 and Fig. 3). On the contrary, for the #2 HLA-A*02:01g-B*18:01g-C*07:01g-DRB1*11:04g haplotype the regions are inversely clustered in three groups, with the southern regions reporting higher frequencies for the haplotype and low numbers of both cases and deaths, whereas central and northern regions show respectively intermediate and low frequencies and progressively increasing reported incidence and mortality of Covid-19 (Figure 4). For this haplotype, the Pearson correlation coefficient among its frequency and Covid-19 incidence varies from − 0.33 (at the 8th of March) to − 0.63 (at the 3rd of May). When considering mortality as the correlated variable the Pearson’s coefficient extends from − 0.28 to − 0.51 (Table 3 and Fig. 4).

**Table 3:** Bivariate correlation analysis among regional haplotypes estimated frequency in the population and COVID-19 incidence and mortality

N°cases/100,000 inhabitants								
Haplotype	8 March		8 April		3May		24 May	
	Correlation	P	Correlation	P	Correlation	P	Correlation	P
HLA-A*01:01g-B*08:01g-C*07:01g-DRB1*03:01g	0.34	0.171	0.75	0.0004***	0.74	0.0004***	0.72	0.0008***
HLA-A*02:01g-B*18:01g-C*07:01g-DRB1*11:04g	− 0.33	0.181	− 0.62	0.006**	− 0.63	0.005**	− 0.61	0.007**
HLA-A*30:01g-B*13:02g-C*06:02g-DRB1*07:01g	− 0.18	0.476	− 0.33	0.183	− 0.33	0.175	− 0.30	0.224
HLA-A*29:02g-B*44:03g-C*16:01g-DRB1*07:01g	0.36	0.141	0.48	0.042*	0.51	0.032*	0.52	0.027*
HLA-A*03:01g-B*07:02g-C*07:02g-DRB1*15:01g	0.17	0.502	0.51	0.033*	0.52	0.028*	0.49	0.038*

N°deaths/100,000 inhabitants								
Haplotype	8 March		8 April		3 May		24 May	
	Correlation	P	Correlation	P	Correlation	P	Correlation	P
HLA-A*01:01g-B*08:01g-C*07:01g-DRB1*03:01g	0.24	0.347	0.52	0.026*	0.57	0.013*	0.57	0.014*
HLA-A*02:01g-B*18:01g-C*07:01g-DRB1*11:04g	− 0.28	0.265	− 0.44	0.065	− 0.50	0.034*	− 0.51	0.032*
HLA-A*30:01g-B*13:02g-C*06:02g-DRB1*07:01g	− 0.19	0.441	− 0.25	0.311	− 0.28	0.257	− 0.29	0.239
HLA-A*29:02g-B*44:03g-C*16:01g-DRB1*07:01g	0.31	0.208	0.36	0.137	0.42	0.083	0.43	0.078
HLA-A*03:01g-B*07:02g-C*07:02g-DRB1*15:01g	0.16	0.538	0.36	0.148	0.40	0.098	0.41	0.094

Correlation = Pearson Correlation Coefficiency. Statistical significance P < 0.05

The haplotypes ranked #4 HLA-A*29:02g-B*44:03g-C*16:01g-DRB1*07:01g and #5 HLA-A*03:01g-B*07:02g-C*07:02g-DRB1*15:01g only show a slight significant correlation with the N° of cases, without a net clustering of the regions in the three areas (north, center, south), whereas the haplotype #3 doesn’t have any correlation (Table 3). Given that single specific alleles are represented in thousand haplotypes in different combinations inside the Italian population, covering a larger percentage of the population, the next step was to determine if the distribution of single HLA-A, -B, -C, -DRB1 alleles of the haplotypes #1 and #2 may in turn overlap with Covid-19 regional distribution. We therefore analysed the estimated frequencies of each allele alone and in all possible double or triple combinations of the four considered HLA-A, -B, -C, -DRB1 loci (Tables 4 and 5). We found that the regional frequencies of the alleles HLA-A*01:01g, HLA-B*08:01g and HLA-DRB1*03:01g were all directly correlated with a higher Covid-19 regional incidence and mortality, with the northern regions having higher frequencies for these alleles. The same was for all the allelic combinations, with a stronger significance for those containing the HLA-B*08:01g and/or the HLA-DRB1*03:01g allele (Table 6) In contrast, the allelic frequencies of HLA-B*18:01, HLA-C*07:01 and HLA-DRB1*11:04 were all inversely related to the number of Covid-19 cases and deaths, having the southern regions higher frequencies and lower incidence and mortality associated to the infection. The same result was observed for all the possible double or triple combinations of the four considered HLA-A, -B, -C, -DRB1 loci (Table 7).

**Table 4:** Frequencies of the single alleles and allelic combinations of the HLA-A, -B, -C, -DRB1 loci of the haplotype HLA-A*01:01g-B*08:01g-C*07:01g-DRB1*03:01g in the Italian population

Frequency (%)							
Regions	HLA-A*01:01g	HLA-B*08:01g	HLA-DRB1*03:01g	HLA-A*01:01g- B*08:01g	HLA-A*01:01g-C*07:01g	HLA-A*01:01g-DRB1*03:01g	HLA-B*08:01g-C*07:01g
Italy	11.53	5.76	9.47	3.45	4.63	2.89	5.16
Abruzzo	11.40	4.34	7.24	2.59	3.73	2.57	3.73
Basilicata	10.28	2.92	7.21	1.33	3.26	1.45	1.35
Calabria	10.37	3.06	5.95	1.38	3.14	0.95	2.34
Campania	11.27	3.97	7.76	1.87	3.62	1.70	3.04
Emilia Romagna	11.84	6.40	8.94	3.61	4.56	2.91	5.76
Friuli Venezia Giulia	11.42	6.49	10.73	4.09	5.25	3.51	5.86
Lazio	11.10	4.70	8.68	2.51	3.66	2.52	4.02
Liguria	12.50	6.33	9.37	3.91	5.03	3.45	5.75
Lombardia	11.84	6.32	9.42	3.74	4.90	3.14	5.78
Marche	12.41	5.22	7.89	3.11	4.49	3.11	4.75
Molise	10.22	3.91	7.17	2.39	3.70	2.61	3.48
Piemonte	11.53	5.79	9.04	3.61	4.68	2.95	5.22
Puglia	11.75	3.56	6.84	1.90	3.46	1.67	2.78
Sardegna	5.33	2.53	21.20	1.36	1.95	1.38	2.26
Sicilia	11.34	3.96	7.02	2.02	3.98	1.80	3.00
Toscana	11.98	6.44	9.78	3.95	5.14	3.36	5.74
Trentino Alto Adige	12.70	8.23	10.67	5.84	6.76	4.76	7.82
Umbria	11.48	6.04	10.12	2.69	3.62	2.84	4.92
Valle D’Aosta	9.02	5.37	7.80	1.22	2.44	1.71	4.39
Veneto	12.45	7.30	10.23	4.82	5.91	3.90	6.87

Frequency (%)						
Regions	HLA-B*08:01g-DRB1*03:01g	HLA-C*07:01g-DRB1*03:01g	HLA-A*01:01g-B*08:01g-C*07:01g	HLA-A*01:01g-B*08:01g-DRB1*03:01g	HLA-A*01:01g-C*07:01g-DRB1*03:01g	HLA-B*08:01g-C*07:01g-DRB1*03:01g
Italy	4.20	4.30	3.40	2.57	2.58	3.80
Abruzzo	3.11	3.21	2.59	2.08	2.08	3.03
Basilicata	1.77	2.20	1.33	1.21	2.08	0.44
Calabria	1.86	2.35	1.36	0.82	2.08	0.60
Campania	2.95	3.11	1.76	1.47	1.51	2.35
Emilia Romagna	4.58	4.65	3.57	2.66	2.67	4.22
Friuli Venezia Giulia	4.97	4.78	4.07	3.15	3.18	4.56
Lazio	3.59	3.78	2.47	2.03	2.09	3.17
Liguria	4.67	4.86	3.88	3.04	3.11	4.33
Lombardia	4.74	4.75	3.68	2.87	2.87	4.37
Marche	3.86	4.05	3.08	2.58	2.69	3.58
Molise	2.61	2.83	2.39	1.74	1.74	2.39
Piemonte	4.16	4.31	3.57	2.66	2.70	3.79
Puglia	2.43	2.50	1.83	1.37	1.32	1.85
Sardegna	1.84	3.32	1.29	1.14	1.11	1.60
Sicilia	2.75	2.87	1.92	1.53	1.49	2.15
Toscana	4.74	4.65	3.88	3.09	3.11	4.38
Trentino Alto Adige	6.23	6.19	5.79	4.50	4.47	6.01
Umbria	4.08	3.41	2.47	2.39	2.17	3.41
Valle D’Aosta	3.90	3.90	1.22	1.22	1.22	2.93
Veneto	5.38	5.45	4.78	3.50	3.52	5.09

**Table 5:** Frequencies of the single alleles and allelic combinations of the HLA-A, -B, -C, -DRB1 loci of the haplotype HLA-A*02.01g-C*07.01g-DRB1*11.04g in the Italian population

Frequency (%)							
Regions	HLA-A*02.01g	HLA-B*18:01g	HLA-C*07:01g	HLA-DRB1*11:04g	HLA-A*02.01g-B*18.01g	HLA-A*02.01g-C*07.01g	HLA-A*02.01g-DRB1*11.04g
Italy	22.82	9.52	17.11	10.07	3.05	4.08	2.59
Abruzzo	24.46	9.97	17.84	12.83	4.47	5.48	4.32
Basilicata	20.69	12.17	19.58	18.05	4.61	5.80	4.73
Calabria	22.25	10.30	21.81	15.27	4.14	5.87	3.99
Campania	21.42	11.01	19.43	14.31	3.64	5.66	3.31
Emilia Romagna	22.63	8.01	16.50	9.57	2.66	4.05	2.37
Friuli Venezia Giulia	24.07	7.66	14.91	8.32	2.27	2.90	2.37
Lazio	22.54	9.12	18.36	11.21	3.17	4.54	3.20
Liguria	22.77	9.54	16.96	9.51	2.81	3.73	2.56
Lombardia	24.19	8.52	15.80	8.98	2.66	3.71	2.58
Marche	22.16	8.86	18.43	10.80	3.55	4.69	3.01
Molise	21.09	10.22	18.48	15.43	3.35	4.35	3.68
Piemonte	22.43	8.99	16.15	9.83	2.81	3.68	2.37
Puglia	20.69	12.08	18.73	16.61	4.20	5.33	4.25
Sardegna	20.46	25.42	24.28	7.33	5.92	5.22	2.04
Sicilia	20.22	10.38	18.52	14.03	3.44	4.74	3.14
Toscana	21.76	8.39	17.15	8.53	2.53	4.04	2.01
Trentino Alto Adige	24.77	6.56	16.24	5.71	2.47	3.54	1.97
Umbria	23.57	8.46	18.13	11.33	2.77	4.91	2.82
Valle D’Aosta	23.90	6.59	15.12	7.07	1.95	1.95	1.46
Veneto	24.73	8.15	16.37	8.14	3.07	3.80	2.30

Frequency (%)							
Regions	HLA-B*18.01g-C*07.01g	HLA-B*18.01g-DRB1*11.04g	HLA-C*07.01g-DRB1*11.04g	HLA-A*02.01g-B*18.01g-C*07.01g	HLA-A*02.01g-B*18.01g-DRB1*11.04g	HLA-A*02.01g-C*07.01g-DRB1*11.04g	HLA-B*18.01g-C*07.01g-DRB1*11.04g
Italy	4.53	3.22	2.81	0.77	1.33	1.28	2.01
Abruzzo	5.41	3.47	3.20	3.13	2.18	2.04	2.09
Basilicata	7.53	6.48	6.17	4.11	3.17	2.95	4.63
Calabria	6.02	4.50	4.72	3.23	2.24	1.93	2.94
Campania	5.66	4.53	4.35	2.80	1.98	1.88	2.76
Emilia Romagna	3.79	2.81	2.42	1.88	1.13	1.06	1.67
Friuli Venezia Giulia	3.82	2.39	2.09	1.40	0.93	0.87	1.60
Lazio	4.50	3.64	3.30	2.25	1.66	1.52	2.10
Liguria	4.15	2.63	2.01	1.84	1.32	1.08	1.60
Lombardia	3.90	2.85	2.27	1.74	1.30	1.22	1.65
Marche	5.44	3.54	3.81	3.09	1.94	2.03	2.64
Molise	6.30	5.76	4.13	3.02	2.35	2.26	3.36
Piemonte	4.39	3.01	2.71	1.94	1.28	1.10	1.89
Puglia	7.16	6.44	5.33	3.40	2.58	2.34	4.54
Sardegna	6.11	2.98	2.13	3.20	1.20	1.13	1.48
Sicilia	5.41	4.47	3.70	2.70	1.81	1.63	2.49
Toscana	3.93	2.21	1.99	1.97	1.05	0.92	1.42
Trentino Alto Adige	3.48	1.75	1.82	1.65	0.79	0.83	1.24
Umbria	3.47	3.86	2.65	2.01	1.52	1.22	1.75
Valle D’Aosta	3.17	1.71	1.22	1.22	0.98	0.49	0.73
Veneto	4.25	2.61	2.40	2.01	1.15	1.27	1.84

**Table 6:** Correlation analysis of the single alleles and allelic combinations of the HLA-A, -B, -C, -DRB1 loci of the haplotype HLA-A*01:01g-B*08:01g-C*07:01g-DRB1*03:01g with N° cases and deaths/100,000 inhabitants

N° cases/100,000 inhabitants								
Haplotype	8 March		8 April		3 May		24 May	
	Correlation	P	Correlation	P	Correlation	P	Correlation	P
HLA-A*01:01g	0.35	0.158	0.68	0.002**	0.67	0.002**	0.64	0.004**
HLA-B*08:01g	0.40	0.097	0.76	0.0002***	0.75	0.0004***	0.72	0.0007***
HLA-DRB1*03:01g	0.28	0.257	0.60	0.009**	0.58	0.012*	0.56	0.016*
HLA-A*01:01g- B*08:01g	0.35	0.152	0.75	0.0003***	0.75	0.0003***	0.73	0.0006***
HLA-A*01:01g-C*07:01g	0.32	0.201	0.71	0.001**	0.71	0.001**	0.68	0.002**
HLA-A*01:01g-DRB1*03:01g	0.32	0.201	0.71	0.001**	0.70	0.001**	0.68	0.002**
HLA-B*08.01g-C*07.01g	0.42	0.080	0.78	0.0002***	0.76	0.0002***	0.74	0.0004***
HLA-B*08.01g-DRB1*03:01g	0.40	0.096	0.76	0.0003***	0.74	0.0004***	0.72	0.0007***
HLA-C*07.01g-DRB1*03:01g	0.43	0.078	0.79	0.0001***	0.78	0.0001***	0.76	0.0002***
HLA-A*01:01g-B*08:01g-C*07:01g	0.35	0.151	0.75	0.0003***	0.76	0.0003***	0.73	0.0005***
HLA-A*01:01g-B*08:01g-DRB1*03:01g	0.33	0.175	0.75	0.0004***	0.74	0.0005***	0.71	0.0009***
HLA-A*01:01g-C*07:01g-DRB1*03:01g	0.30	0.232	0.72	0.0008***	0.71	0.0009***	0.68	0.002**
HLA-B*08.01g-C*07.01g-DRB1*03:01g	0.41	0.094	0.75	0.0003***	0.74	0.0004***	0.72	0.0007***

N° deaths/100,000 inhabitants								
Haplotype	8 March		8 April		3 May		24 May	
	Correlation	P	Correlation	P	Correlation	P	Correlation	P
HLA-A*01:01g	0.26	0.289	0.53	0.025*	0.56	0.015*	0.56	0.015*
HLA-B*08:01g	0.31	0.216	0.54	0.022*	0.58	0.011*	0.58	0.011*
HLA-DRB1*03:01g	0.21	0.399	0.38	0.121	0.42	0.080	0.43	0.078
HLA-A*01:01g- B*08:01g	0.25	0.317	0.52	0.026*	0.58	0.012*	0.58	0.012*
HLA-A*01:01g-C*07:01g	0.22	0.379	0.49	0.038*	0.54	0.020*	0.54	0.022*
HLA-A*01:01g-DRB1*03:01g	0.20	0.419	0.48	0.042*	0.53	0.024*	0.52	0.025*
HLA-B*08.01g-C*07.01g	0.32	0.198	0.56	0.016*	0.61	0.008**	0.60	0.008**
HLA-B*08.01g-DRB1*03:01g	0.31	0.206	0.55	0.019*	0.59	0.010*	0.59	0.010*
HLA-C*07.01g-DRB1*03:01g	0.33	0.177	0.58	0.011*	0.63	0.005**	0.63	0.005**
HLA-A*01:01g-B*08:01g-C*07:01g	0.25	0.315	0.52	0.025*	0.58	0.011*	0.58	0.012*
HLA-A*01:01g-B*08:01g-DRB1*03:01g	0.23	0.350	0.52	0.028*	0.57	0.014*	0.56	0.015*
HLA-A*01:01g-C*07:01g-DRB1*03:01g	0.21	0.408	0.50	0.036*	0.54	0.020*	0.54	0.021*
HLA-B*08.01g-C*07.01g-DRB1*03:01g	0.31	0.216	0.54	0.019*	0.59	0.010*	0.59	0.010*

**Table 7:** Correlation analysis of the single alleles and allelic combinations of the HLA-A, -B, -C, -DRB1 loci of the haplotype HLA-A*02.01g-C*07.01g-DRB1*11.04g with N° cases and deaths/100,000 inhabitants

N° cases/100,000 inhabitants								
Haplotype	**8 March**		**8 April**		**3 May**		**24 May**	
	**Correlation**	**P**	**Correlation**	**P**	**Correlation**	**P**	**Correlation**	**P**
HLA-A*02:01g	0.33	0.175	0.59	0.009**	0.56	0.015*	0.55	0.019*
HLA-B*18:01g	− 0.39	0.113	− 0.68	0.002**	− 0.64	0.004**	− 0.62	0.006**
HLA-C*07:01g	− 0.47	0.050	− 0.71	0.001**	− 0.72	0.001**	− 0.72	0.001**
HLA-DRB1*11:04g	− 0.41	0.093	− 0.75	0.0003***	− 0.75	0.0004*	− 0.72	0.001**
HLA-A*02:01g-B*18:01g	− 0.38	0.122	− 0.61	0.008**	− 0.60	0.009**	− 0.59	0.010*
HLA-A*02:01g-C*07:01g	− 0.41	0.095	− 0.66	0.003**	− 0.68	0.002**	− 0.68	0.002**
HLA-A*02:01g-DRB1*11:04g	− 0.39	0.110	− 0.66	0.003**	− 0.66	0.003**	− 0.64	0.004**
HLA-B*18.01g-C*07.01g	− 0.37	0.128	− 0.64	0.004**	− 0.63	0.005**	− 0.61	0.007**
HLA-B*18.01g-DRB1*11.04g	− 0.35	0.150	− 0.69	0.002**	− 0.69	0.002**	− 0.67	0.002**
HLA-C*07.01g-DRB1*11.04g	− 0.36	0.137	− 0.67	0.002**	− 0.67	0.002**	− 0.66	0.003**
HLA-A*02:01g-B*18:01g-C*07:01g	− 0.37	0.132	− 0.63	0.005**	− 0.64	0.004**	− 0.63	0.005**
HLA-A*02:01g-B*18:01g-DRB1*11:04g	− 0.35	0.150	− 0.65	0.003**	− 0.66	0.003**	− 0.64	0.005**
HLA-A*02:01g-C*07:01g-DRB1*11:04g	− 0.29	0.238	− 0.60	0.009**	− 0.61	0.007**	− 0.60	0.009**
HLA-B*18.01g-C*07.01g-DRB1*11.04g	− 0.32	0.190	− 0.61	0.008**	− 0.61	0.008**	− 0.59	0.009**

N° deaths/100,000 inhabitants								
Haplotype	8 March		8 April		3 May		24 May	
	Correlation	P	Correlation	P	Correlation	P	Correlation	P
HLA-A*02:01g	0.30	0.222	0.45	0.063	0.46	0.057	0.46	0.056
HLA-B*18:01g	− 0.27	0.287	− 0.47	0.048*	− 0.49	0.037*	− 0.48	0.042*
HLA-C*07:01g	− 0.40	0.098	− 0.56	0.016*	− 0.62	0.006**	− 0.62	0.006**
HLA-DRB1*11:04g	− 0.31	0.206	− 0.55	0.017*	− 0.60	0.008**	− 0.60	0.008**
HLA-A*02:01g-B*18:01g	− 0.31	0.212	− 0.45	0.060	− 0.49	0.037*	− 0.49	0.038*
HLA-A*02:01g-C*07:01g	− 0.33	0.185	− 0.51	0.031*	− 0.57	0.013*	− 0.58	0.012*
HLA-A*02:01g-DRB1*11:04g	− 0.28	0.263	− 0.47	0.050	− 0.52	0.025*	− 0.53	0.025*
HLA-B*18.01g-C*07.01g	− 0.32	0.201	− 0.47	0.048*	− 0.51	0.030*	− 0.51	0.029*
HLA-B*18.01g-DRB1*11.04g	− 0.27	0.270	− 0.51	0.032*	− 0.56	0.015*	− 0.57	0.014*
HLA-C*07.01g-DRB1*11.04g	− 0.31	0.218	− 0.50	0.036*	− 0.55	0.017*	− 0.56	0.015*
HLA-A*02:01g-B*18:01g-C*07:01g	− 0.33	0.177	− 0.47	0.047*	− 0.53	0.024*	− 0.54	0.022*
HLA-A*02:01g-B*18:01g-DRB1*11:04g	− 0.28	0.267	− 0.46	0.055	− 0.52	0.028*	− 0.52	0.027*
HLA-A*02:01g-C*07:01g-DRB1*11:04g	− 0.25	0.320	− 0.42	0.083	− 0.48	0.042*	− 0.49	0.038*
HLA-B*18.01g-C*07.01g-DRB1*11.04g	− 0.27	0.284	− 0.45	0.062	− 0.50	0.036*	− 0.50	0.034*

## Discussion

In the present study, through a geographical epidemiological analysis, we observed that there are significative regional differences in the frequency of the two most common HLA haplotypes in the Italian population among the northern, central and southern regions with HLA-A*01:01g-B*08:01g-C*07:01g-DRB1*03:01g (ranked #1 at the national level) showing a decreasing frequency gradient and HLA-A*02:01g-B*18:01g-C*07:01g-DRB1*11:04g (ranked #2) an increasing frequency gradient from North to South. The geographical distribution of these haplotypes overlaps with that of Covid-19 in Italy, being linearly correlated in a positive/direct way for the haplotype #1 and in a negative/inverse way for the haplotype #2. This means that a high incidence and mortality was observed in the northern regions where the population has high frequency values of the haplotype HLA-A*01:01g-B*08:01g-C*07:01g-DRB1*03:01g and all the allelic combinations of the four considered HLA-A, -B, -C, -DRB1 loci, containing at least one of these alleles, particularly those with the B*08:01g and DRB1*03:01g polymorphism, suggestive of potential ‘susceptibility’ to the disease. On the contrary, a low incidence and mortality for Covid-19 was observed in the central-southern regions with high frequency values of the haplotype HLA-A*02:01g-B*18:01g-C*07:01g-DRB1*11:04g and of its alleles B*18:01g, C*07:01g and DRB1*11:04g in all their possible combinations containing at least one of such alleles, suggestive of potential ‘protection’ from the infection. Hence, the population of central-southern Italy that shows the highest prevalence of the protective haplotype HLA-A*02:01g-B*18:01g-C*07:01g-DRB1*11:04g and its allelic combinations and, at the same time, the lowest frequencies of the disadvantageous haplotype HLA-A*01:01g-B*08:01g-C*07:01g-DRB1*03:01g and its allelic combinations, could be genetically shielded from Covid-19. Such findings are only descriptive in nature and need to be validated through retrospective observational case–control studies on Covid-19 patients typed for HLA comparing the frequencies of the potential ‘protective’ and ‘unfavourable’ HLA haplotypes and alleles highlighted in the general Italian population with those observed in the Covid-19 patients cohort, in order to define such HLA polymorphisms as a factor effectively associated to the disease susceptibility as already done for other viral infections, communicable diseases and autoimmune pathologies [15–19]. However, also in these pathologies such geographical epidemiological approaches have given important clues to identify sub-populations most at risk of susceptibility to the infection also taking into account as a susceptibility parameter HLA specific alleles and haplotypes [13].

To the best of our knowledge, this is the first study that estimated, through a population frequency analysis, the potential association of specific HLA alleles and haplotypes with the incidence and mortality of Covid-19. Although the primary scope of a bone marrow registry is to increase the possibilities to find allogenic compatible donors for transplants, it is also a unique source of precious HLA data from the widest and most representative sample available at the national level, which makes it possible to reliably estimate haplotypes frequencies in a given population and carry out association studies in many disease contexts. We conducted our study on a large sample of 104,135 subjects typed at high resolution four-digit level, subdivided in the 20 Italian regions, with a regional sample size adequately statistically representative of the resident population for each region [20].

Our study is the first to propose HLA as a susceptibility marker to SARS-CoV-2 infection and highlight its potential impact on the epidemic trend within a specific country, Italy, that has been hit particularly hard. However, similar associations may also be observed within other countries, bringing to light common genetic patterns or new country-specific protective or unfavourable HLA polymorphisms, that could explain some of the differences observed in the epidemic between one country and another. Such geographical epidemiological studies, conducted at the general population level, need to be confirmed in Covid-19 patients’ cohorts of asymptomatic, mildly symptomatic, severely affected individuals to draw fundamental conclusions with important implications not only at the epidemiological level but also at the clinical one. Indeed, particular HLA haplotypes/alleles could be associated with a stronger immune response and hence a better host response to the virus. Some useful information can also be inferred by previous researches on SARS and MERS, where it has been reported that several HLA polymorphisms are associated to SARS susceptibility (HLA-B*46:01, HLA-B*07:03, HLA-DRB1*12:02 and HLA-Cw*08:01) [25–27]. On the contrary the allelotypes HLA-DR*03:01, HLA-Cw*15:02 and HLA-A*02:01 seem to be protective from SARS infection [28]. HLA-DRB1*11:01 and HLA-DQB1*02:02 are related to MERS-CoV infection susceptibility [29]. On these premises, it is conceivable that several HLA associations could be unfavourable or protective also for the course of Covid-19 infection.

Very recent works employed different bioinformatic approaches to predict the best SARS-CoV-2 derived B and T cell epitopes and their associated HLA alleles, that may help to design effective vaccines and find protective antibodies [30–35]. Employing HLA binding affinity prediction tools, it has been observed that HLA-A and HLA-C alleles exhibited the relatively most and least capacity to present SARS-CoV-2 antigens, respectively. However, depending on the specific study and the bioinformatic approach used, the best and worse predicted presenters of conserved peptides reported are not the same. We found that the alleles analysed in our study are present in the database recently made available by Nguyen et al., that reports the list of 32,257 8- to 12-mers peptides from the SARS-CoV-2 proteome and their binding affinity to 145 different HLA A, B, C alleles, predicted by bioinformatic tools [30]. In particular, all the alleles pointed out in our study have been predicted to have an overall good capacity to present viral peptides, independently of their potential correlation with Covid-19 regional incidence and mortality, with HLA-A*02:01 being the best (1062 total peptides, 268 with a very high binding affinity < 50 nM), followed by HLA-B*08:01 (225 total, 25 high affinity), HLA-A*01:01 (183 total, 44 high affinity), HLA-B*18:01 (101 total, 12 high) and HLA-C*07:01 (44 total, 4 high) (Additional file 1: Fig. S1) [30].

It is important to note that all the bioinformatic predictions made on SARS-CoV-2 epitopes and their HLA binding, have the limit to be exclusively theoretical and thus need to be experimentally validated in in vitro binding assays and in the ability to effectively elicit T and B cell mediated responses. Indeed, it is widely recognised that antigenicity, immunogenicity and, for T cells, the TCR avidity to the antigen/HLA and hence the functional immune responses elicited, are not directly related with the peptide binding affinity [36, 37]. No information is available to date regarding the binding of HLA II molecules, whose polymorphic variants could play a relevant role in orchestrating a functional adaptive immune response.

Undoubtedly, the method of analysis used in our study presents some limits and could be affected by an inevitable selection bias, since it takes into consideration the region of birth of the typed individuals but not the region of residence, whereas data about Covid-19 infections are reported per region where the infection occurred, independently of birthplace. However, we can reasonably exclude the influence of migration flows (that in Italy are historically directed from the southern regions to the northern) on the regional frequencies used in our computations, since they are equivalent to those from previous studies with information concerning both the region of birth and residence and so, thanks to the large dimension of the regional subgroups analysed, independent from the migratory movements [38, 39]. The information about Covid-19 cases and deaths relies on public resources, daily updated on the basis of laboratory analysis of swabs tested positive for the virus by RT-PCR at the regional accredited centers, following confirmatory testing by the Italian National Institute of Health in Rome. As above reported, these values could have been underestimated for reasons depending on several factors like a stringent testing policy, limited to severely affected symptomatic individuals, that excluded from testing the bulk of asymptomatic ones, shortage of testing materials in the peak of the emergency, limited access to overcrowded hospital facilities, to name just a few. Noteworthy, a higher overall mortality rate than previous years has been observed in Nembro, a little town of Lombardia region, indicative of both direct and indirect disease burden and has been also highlighted by a recent report published by Italian National Institute for Social Security [5, 6].

Apart from the epidemiological value in tracing the distribution of Covid-19 and understanding its immunopathogenesis, the identification of specific HLA haplotypes as potential risk, susceptibility or protective biomarkers, can be of great help in stratifying the population, in order to identify those patients more at risk to develop a severe infection, thus allowing to adopt proper preventive strategies and early intervention measures.

It is important to note that the HLA region is known for its linkage disequilibrium, therefore, other genes very near to HLA could be eventually responsible for the association with Covid-19 regional distribution. Genetic polymorphisms in the HLA locus or in other genes encoding key components of the immune-inflammatory response observed in SARS-CoV-2 infection (KIR receptors, inflammasome components, cytokines and chemokines like CXCL10) may help to explain the high variable spectrum of disease manifestations, progression and outcome (from asymptomatic, to mild-moderately symptomatic and severely affected patients requiring intensive care and respiratory support).

With this in mind, even though the collected knowledge is still limited to few studies, some susceptibility markers other than HLA have been proposed for Covid-19. An association with ABO blood antigens has been observed in a cohort of Chinese patients, with the type A and 0 being respectively at highest and lowest risk to be infected, as previously been reported for other viral infections [40]. This observation was confirmed in a genomewide study on Spanish and Italian patients’ cohorts. Indeed, a skewing of ABO blood antigens distribution among Covid-19 patients who suffered from respiratory failure was reported, whereas no significant association was found between HLA polymorphisms in Covid-19 patients and respiratory failure (oxygen supplementation or mechanical ventilation) [41]. To the best of our knowledge this is the only study available to date that takes into account the association of HLA polymorphisms and Covid-19 severity, but it is important to note that it was performed in a limited Italian population, including only patients from Lombardia region, without taking into account geographical patterns of HLA distribution. Genetic polymorphisms of key genes of the virus entry machinery (*Ace2, Tmprss2, CtsB, and CtsL*) or of the inflammatory/immune response (*e.g.* cytokines and their receptors) or epigenetic mechanisms may influence virus susceptibility and the severity/outcome of the infection among different individuals and populations, too [42, 43]. A novel susceptibility locus containing a cluster of six genes (SLC6A20, LZTFL1, CCR9, FYCO1, CXCR6, and XCR1) on chromosome 3p21.31, most of whose involved in the regulation of inflammatory and immune response, has been indeed found [41].

We recognize that other factors, *e.g.* climatic differences, pollution, lockdown effect that limited the diffusion from North to South, could be responsible alone or in combination with genetic factors for the different Covid-19 infection rates among Italian regions. Our reported potential association of two haplotypes with the differential regional incidence and mortality for Covid-19 in Italy may explain, from the point of view of the genetic diversity of the Italian population, why the epidemic hit the northern regions so hard and instead had a small impact on those of the central-south, a figure which cannot be explained on the basis of population, urban density, movements to and from large urban and industrial areas, pollution or climate alone. Indeed, several central and southern metropolitan areas like those of Rome, Naples, Bari, Palermo (respectively located in Lazio, Campania, Puglia, Sicilia) have an urban density comparable or even higher (Naples) than Milan and Lombardia, atmospheric emissions of PM10, PM2.5 and NO_2_ levels above threshold, and high flows of mobility through public transports [44]. Furthermore, the climatic variations in Italy are very limited and not comparable to those occurring in wider countries like China, US or Brasil [45].

Our correlation analysis among HLA regional frequencies and Covid-19 cases/deaths numbers, having been carried out at different times over the epidemic, also takes into account the potential effects elicited by the displacement of thousands of off-site students and workers from the northern (mainly Lombardia, the fire of the epidemic) to the southern regions (Campania, Puglia, Calabria, Sicilia), which occurred in two large waves, the night before the start of the lockdown (the 9th of March, totally uncontrolled) and at the end of the lockdown (after the 3rd of May, with some monitoring from region to region). These uncontrolled exoduses and especially the first one, although occurring in a phase of mobility restrictions and contact reduction, could have caused the epidemic to break out in the southern Italian regions, which instead did not occur and which makes the hypothesis of a protective genetics even more plausible in the populations of central-southern Italy.

Genetic variations and HLA polymorphisms alone cannot help to understand other significant features of Covid-19, like the higher mortality observed in men *vs* women (2.8% vs 1.7%) or the higher morbidity and mortality in old *vs* young people [46–48]. However, it is fundamental to take into account that significant differences at the immunological level exist among these groups and such differences could be dependent on HLA polymorphisms and, overall, on the genetic, hormonal and metabolic background. Indeed, HLA genes are involved in the decline of anti-viral response mediated by T cells that is observed with aging.

## Conclusion

Our study proposes for the first time that some HLA polymorphisms in the Italian population may be potentially associated to the different regional incidence and mortality for Covid-19, likely activating a better and more powerful antiviral response, with central-southern regions being most protected from the epidemic. Such evidence, obtained at the general population level, needs to be confirmed in retrospective case–control studies on wide cohorts of Covid-19 patients from all the Italian regions in order to define HLA polymorphisms as a factor involved in disease susceptibility. Moreover, since the bioinformatic predictions on HLA-viral peptides binding affinity alone are of limited functional significance, it is fundamental to identify through proper in vitro and in vivo studies, if such HLA genetic loci are effectively associated to the induction of a protective T and B-cell mediated antiviral immunity. Research efforts aimed to explore genetic associations with the immune response in Covid-19 could be particularly useful both at the epidemiological and clinical level, to identify patients most at risk to develop severe complications, that should hence have priority to vaccination access, when it will be available, and to evaluate the differential efficacy of the vaccination in subjects with different HLA genetic background. HLA typing, that can be easily done through cost-efficient methodologies, also along with Covid-19 testing, should hence be envisaged and encouraged at the clinical level and by policy makers through the creation of a national network that may collect DNA samples from patients from all regions.

## Supplementary information

**Additional file 1: Table S1.** Regional statistics relative to the Italian population (2019 data from ISTAT). **Figure S1** The histograms report the number of peptides with high binding affinity (< 50 nM) that have been predicted to bind to HLA-A, -B, and -C most frequent alleles worldwide by Nguyen et al. 2020 (public database at https://github.com/pdxgx/covid19/tree/master/supporting_data).

## Data Availability

Most of the data used in this study are freely available from the source cited. Some data are available upon reasonable request from the corresponding authors.

## Abbreviations

ARSD: Acute respiratory distress syndrome
HLA: Human leukocyte antigen
HIV: Human immunodeficiency virus
HBV: Human hepatitis B virus
HCV: Human hepatitis C virus
HPV: Human papilloma virus
INPS: National Institute for Social Security
ISS: Istituto Superiore di Sanità
ISTAT: Italian National Institutes of Statistics
RT-PCR: Reverse transcriptase–polymerase chain reaction
