# Supplementary material for: Correlation of the two most frequent HLA haplotypes in the Italian population to the differential regional incidence of Covid-19
Source: J Transl Med. 2020 Sep 15;18:352. doi: 10.1186/s12967-020-02515-5 (PMC7491019; doi:10.1186/s12967-020-02515-5)
Supplement: Supplementary file 1 — Additional file 1: Table S1. Regional statistics relative to the Italian population (2019 data from ISTAT). Figure S1 The histograms report the number of peptides with high binding affinity (< 50 nM) that have been predicted to bind to HLA-A, -B, and -C most frequent alleles worldwide by Nguyen et al. 2020 (public database at https://github.com/pdxgx/covid19/tree/master/supporting_data). [file 12967_2020_2515_MOESM1_ESM.docx]

**Table S1. Regional statistics relative to the Italian population (2019 data from ISTAT)**

|  | **Resident population** | **% Resident population** | **Area (Km^2^)** | **Inhabitants/Km^2^** |
| --- | --- | --- | --- | --- |
| Italy | 60359546 | 100 | 302073.63 | 199.82 |
| Abruzzo | 1311580 | 2.17 | 10831.84 | 121.09 |
| Basilicata | 562869 | 0.93 | 10073.32 | 55.88 |
| Calabria | 1947131 | 3.23 | 15221.9 | 127.92 |
| Campania | 5801692 | 9.61 | 13670.95 | 424.38 |
| Emilia Romagna | 4459477 | 7.39 | 22452.78 | 198.62 |
| Friuli Venezia Giulia | 1215220 | 2.01 | 7924.36 | 153.35 |
| Lazio | 5879082 | 9.74 | 17232.29 | 341.17 |
| Liguria | 1550640 | 2.57 | 5416.21 | 286.30 |
| Lombardia | 10060574 | 16.67 | 23863.65 | 421.59 |
| Marche | 1525271 | 2.53 | 9401.38 | 162.24 |
| Molise | 305617 | 0.51 | 4460.65 | 68.51 |
| Piemonte | 4356406 | 7.22 | 25387.87 | 171.59 |
| Puglia | 4029053 | 6.68 | 19540.9 | 206.19 |
| Sardegna | 1639591 | 2.72 | 24100.02 | 68.03 |
| Sicilia | 4999891 | 8.28 | 25832.39 | 193.55 |
| Toscana | 3729641 | 6.18 | 22987.04 | 162.25 |
| Trentino Alto Adige | 1072276 | 1.78 | 13605.5 | 78.81 |
| Umbria | 882015 | 1.46 | 8464.33 | 104.20 |
| Valle D'Aosta | 125666 | 0.21 | 3260.9 | 38.54 |
| Veneto | 4905854 | 8.13 | 18345.35 | 267.42 |

**Figure S1**

The histograms report the number of peptides with high binding affinity (<50 nM) that have been predicted to bind to HLA-A, B, and C most frequent alleles worldwide by Nguyen et al. 2020 (public database at https://github.com/pdxgx/covid19/tree/master/supporting_data)

**

**

**



**
